# Hypervigilance or avoidance of trigger related cues in migraineurs? - A case-control study using the emotional stroop task

**DOI:** 10.1186/1471-2377-11-141

**Published:** 2011-11-05

**Authors:** Anne-Katrin Puschmann, Claudia Sommer

**Affiliations:** 1 Department of Neurology, University of Wuerzburg, Josef-Schneider-Str. 11, 97080 Wuerzburg, Germany; 2 Research Training Group „Emotions", Department of Psychology I, University of Wuerzburg, Marcusstr. 9-11, 97074 Wuerzburg, Germany

## Abstract

**Background:**

"Negative affect" is one of the major migraine triggers. The aim of the study was to assess attentional biases for negative affective stimuli that might be related to migraine triggers in migraine patients with either few or frequent migraine and healthy controls.

**Methods:**

Thirty-three subjects with frequent migraine (FM) or with less frequent episodic migraine, and 20 healthy controls conducted two emotional Stroop tasks in the interictal period. In task 1, general affective words and in task 2, pictures of affective faces (angry, neutral, happy) were used. For each task we calculated two emotional Stroop indices. Groups were compared using one-way ANOVAs.

**Results:**

The expected attentional bias in migraine patients was not found. However, in task 2 the controls showed a significant attentional bias to negative faces, whereas the FM group showed indices near zero. Thus, the FM group responded faster to negative than to positive stimuli. The difference between the groups was statistically significant.

**Conclusions:**

The findings in the FM group may reflect a learned avoidance mechanism away from affective migraine triggers.

## Background

The reported prevalence of chronic migraine in the population is 1.4-2.2.% [1]. Patients with episodic migraine have an annual risk of 2,5% to develop chronic migraine [2]. An intermediate headache frequency of 6 to 9 days per month and even more a critical frequency of 10 to 14 headache days per month increase the risk for chronicity [3]. Further risk factors are obesity, stressful life events, snoring, and overuse of certain classes of medication. Up to 90% of migraine patients are able to name trigger factors like emotional stress, sleep deprivation, visual triggers (e.g. flickering lights) or hormonal changes that precipitate their attacks [4,5]. Triggers are equally named by patients with episodic and chronic migraine [6]. The mode of action of these migraine triggers is as yet unknown. Several authors proposed trigeminal signaling mechanisms that sensitize certain brain areas, resulting in general sensitization of meningeal nociceptors and subsequently migraine pain [7,8]. It has also been suggested that the interaction of several triggers leads to migraine attacks [9,10].

An attentional bias towards pain-related cues, i.e. selective attention to pain-related information, is one of the psychological variables that are thought to be involved in turning an episodic into a chronic pain disorder [11,12]. The emotional Stroop paradigm is a means to assess this bias. In this task subjects have to name the color of emotionally relevant stimuli as fast as possible while ignoring the affective content of the stimuli. The underlying theory assumes that threatening (pain-related) stimuli draw the subjects' attention, and thus increase the latencies for colour naming compared to neutral stimuli or positive stimuli [13,14]. The opposite effect can occur when the affective content of the stimuli is avoided and color naming of negative stimuli therefore is speeded up [15]. A recent metanalysis identified five studies using an emotional Stroop task with pain-related words that found an attentional bias towards sensory and affective pain words in chronic pain patients [16]. Similar results were found in a study using personalized pain words [17]. However, no such attentional bias was found in chronic headache patients [18]. Thus, until now, there is no evidence of attentional biases in the Stroop task in patients with migraine. Other studies investigated chronic pain patients using the so called visual probe task and failed to find the expected attentional bias [19-21]. In contrast, Liossi and collegues [22] found selective attention towards pain related words in a group of headache patients.

We hypothesized that for migraine patients trigger related stimuli rather than pain related stimuli are threatening and thus cause an attentional bias. As most of the patients associate their attacks to trigger factors as reported above, it is likely that these trigger factors over time develop a threatening character for the patients. Therefore, we conducted two emotional Stroop tasks, one with general affective words and the second with social affective cues. In the latter, pictures of affective faces are used due to their higher ecological validity [23]. An attentional bias towards negative affective stimuli was expected in both tasks for a group of subjects with very frequent migraine (FM, thus at higher risk for or already transformed to chronic migraine) compared to subjects with less frequent, episodic migraine (EM) and healthy controls. We hypothesized that FM patients should be faster in colour naming of neutral or positive stimuli than of negative stimuli, which would be reflected in higher stroop indices.

## Methods

### Participants

33 subjects with migraine diagnosed according to the diagnostic criteria of the International Headache Society (IHS, 2004) were recruited via the Pain and Headache Clinic of the Department of Neurology at the University hospital, Würzburg. The control group consisted of 20 age- and sex-matched healthy persons without migraines or frequent headaches, recruited via newspaper announcements. Exclusion criteria were: other pain, neurological or inflammatory acute or chronic diseases, antidepressive, anticonvulsive, neuroleptic or anti-inflammatory medication, and symptomatic medication like triptans or NSAIDs in the 48 hours preceding the day of testing. Patients with only menstrual migraine were excluded. Each subject gave written informed consent. Approval of the ethics comitee of the university of Wuerzburg was granted. Subjects received a small compensation for participation. German was the prime language used by all participants. All had normal or corrected-to-normal vision. None were under the influence of alcohol or drugs at the time of testing, and none had consumed caffeine within the three hours prior to testing. Information about alcohol, drug, and caffeine consumption was obtained through self report (i.e. no blood or urine indices were conducted for confirmation). Patients were tested interictally at least 48 hours after the last migraine attack and all were headache free at the time of testing. One limitation of conducting this type of study in a population of patients with frequent headache is that some of them might already be in a pre-ictal stage. They were asked to name their personal migraine trigger factors.

Patients were divided into two groups according to their monthly migraine and headache frequency: FM, when the headache frequency was at least 10 headache days per month and the monthly frequency of migraine attacks was at least four (n = 16), and EM, when the headache frequency was below 10 headache days per month and the monthly attack frequency was below four (n = 17, Table 1). This division was chosen instead of following the IHS definition for episodic and chronic migraine, because we wanted to include those patients at high risk for migraine chronification [3].

**Table 1:** Demographic data and headache parameters for all three groups.

	Group		
	Co	EM	FM
*n*	20	17	16
female	90.5%	85%	100%
age (*M, SD)*	39.8 (10.5)	41.35 (11.87)	43.4 (13.3)
migraine duration in years (*M, SD)*	no headaches	22 (12)	27 (15)
migraine attacks per month (*M, SD)*	no headaches	1.7 (1.2)	6.4 (2.7)
headache days per month (*M, SD)*	no headaches	4.5 (2.2)	17.2 (6.37)
prophylaxis - betablockers ^a^	5%	35%	16%
*triggers ^b^*	no headaches		
stress		85%	81%
hormones		30%	44%
weather		50%	75%
visual triggers		5%	50%
sleep problems		55%	56%

Co: healthy controls; EM: episodic migraine patients; FM: frequent migraine patients.

*^a ^Most frequently named trigger factors of a list of 20*.

Table 1 displays descriptive data and the headache parameters of the participants. The three groups did not differ regarding their age (*M=*41 years, *SD=*12). The two migraine groups did not differ regarding time since migraine onset (disease duration; *M=*15, *SD=*15). The migraine groups differed significantly with respect to the number of headache days per month (EM: *M=*4.5, *SD=*2.2; FM: *M=*17.2; *SD=*6.4; *t*(18)=-7.58, *p *< .001) and to the monthly number of migraine attacks (EM: *M=*1.6, *SD=*1.2; FM: *M=*6.4, *SD=*2.8; *t*(20)=-6.30, *p *< .001). They did not differ in the frequency of migraine with aura (EM: 37%, FM: 59%; *χ*^2^(1) = 1.72, *p = *.316). The majority of the patients named "psychosocial stress" as an important trigger factor for their attacks (83%). Hormonal changes, weather and sleep problems were also named by nearly half of the patients.

### Self-report instruments

Participants completed several self-report questionnaires. To assess general disability due to headaches subjects filled in the German version of the Migraine Disability Assessment Score (MIDAS; [24]). Trait anxiety was assessed using the German trait version of the State Trait Anxiety Inventory (STAI-T; [25]), a 20-item likert-scaled anxiety questionnaire. Depressive symptoms were measured using the German revised Beck Depression Inventory (BDI-II; [26,27]). Additionally, the Penn State Worry Questionnaire was used to learn about the occurrence of worries in the participants (PSWQ; [28]).

### Experiment

Both tasks were computerized versions of the modified Stroop test [13] with emotional stimuli (emotional Stroop test). Since our group of our participants consisted mainly of middle aged women with little experience in computer usage, we chose this relativly simple task to make sure they would be able to perform it without problems or misunderstandings after a set of practice trials with neutral stimuli.

#### Task 1: Emotional Stroop test - words

To assess a possible attentional bias towards negative emotional stimuli, in the first experiment 36 affective nouns from the validated Berlin Affective Word List [29] were used, 12 for each valence category (negative, neutral, positive) (Table 2). Words were matched according to length and frequency. The experiment was programmed using the software Presentation^® ^by Neurobehavioral Systems. Stimuli were presented in random order with a size of 36 pt. Each word was presented in the colors red, green, and blue on a black background in the centre of a 17"-computer screen. In total the experiment consisted of 108 trials. Participants were instructed to name the color of the words by pressing the button with the correct color on a colored keyboard as fast as possible while ignoring word content. After pressing the button the word disappeared. If the button was not pressed, words disappeared after 2000 ms. After an inter trial interval of 500 ms the next word appeared. The experiment was preceded by a 40-trial practice part with neutral words. The color naming latencies for each word and also the number of incorrect responses were recorded for each participant.

**Table 2:** Words from the Berlin Affective Word List (translated).

Valence category		
Negative	Neutral	Positive
murder	chin	fortune
fear	base	fun
army	stone	kiss
poison	figure	friend
homicide	mood	pleasure
slave	hall	blossom
betrayal	area	humor
panic	guarantee	love
prison	reaction	trust
eradication	semester	laughter
threat	interview	amusement
agony	influence	health

#### Task 2: Emotional Stroop test - faces

To assess a possible attentional bias towards social affective cues, in the second experiment 81 pictures of affective faces were used as stimuli. Pictures were taken from the Karolinska Directed Emotional Faces (KDEF; [30]). Each valence category (negative: angry faces, neutral: neutral faces, positive: happy faces) consisted of 27 pictures. Each picture was displayed in black and white and had a frame of red, green, or blue. In total, the experiment consisted of 81 trials which were presented in random order on a black background at a 17"-computer screen. There were three sets of trials to prevent systematic errors due to colour. In each set pictures were only presented once, but each time with different colors. The sets were allocated equally to the participants. Similarly to the previous experiment participants were instructed to name the color of the frame as fast as possible through pressing the corresponding colored button on the keyboard while ignoring picture content. Between the trials a fixation cross was displayed for 500 ms. Maximum possible response time was 2000 ms. Reaction times as well as errors were recorded.

### Statistical analyses

Statistical analyses were conducted using the software SPSS 17.02. Two emotional Stroop indices (ESI) were calculated. The first index compares the color naming latencies of negative stimuli with the latencies of neutral stimuli (ESI-N), as done in the anxiety [31] and pain literature [12]. ESI-N is calculated using the following formula: *ESI-N = RTnegative - RTneutral *. The second index compares the response latencies of negative stimuli with the latencies of positive stimuli (ESI-P), as described in the original emotional Stroop study [13]. ESI-P is calculated as follows: *ESI-P = RTnegative - RTpositive*. RTnegative are the mean color naming latencies for negative words or faces, RTneutral are the mean color naming latencies for neutral words or faces, and RTpositive are the mean color naming latencies for positive words or faces. All indices are displayed in milliseconds. One-sample Student's *t*-tests were calculated to obtain informations about the difference of the indices from zero. Positive indices indicate slower reactions to negative stimuli than to neutral or positive stimuli; negative indices indicate faster reactions to negative stimuli. To compare the three groups, one-way analyses of variances were calculated for each index and experiment separately. A priori contrasts were set to specify the group differences according to the hypotheses. Alpha-level was set at *p *< .05. In addition, Pearson correlations between the indices and headache parameters as well as the self-report scores were calculated.

## Results

### Data reduction and errors

Fifty-three participants completed the above described tests. In total 2.3% of the data were excluded due to reaction errors. There were no group differences in the occurrence of errors (*p*>.05). Data with latencies smaller than 200 ms and above 1200 ms were excluded as outliers (3.5%).

### Self reports

Table 3 shows the mean scores of the self-report questionnaires for each group. Regarding the disability due to migraine and headaches the two patient groups differed significantly (*F*(32,1) = 12.67, *p=*.001) with the FM group exhibiting a threefold higher MIDAS score than the EM group. 67% of the FM patients reached grade IV with "severe disability" in contrast to 16% of the EM patients. The three groups furthermore differed regarding their BDI-II scores (*F*(2,50) = 3.64; *p=*.033) with the FM group exhibiting the highest score (*M=*12, *SD=*9). 20% of the FM group had moderate to severe depressive symptoms in comparison to 11% in the EM group and 5% in the control group, respectively. With respect to trait anxiety the STAI-T scores of the three groups did not differ (*F*(2,50) = 3.01, *p=*.058). Analyses of the PSWQ scores revealed a significant main effect for group (*F*(2,50) = 5.49, *p=*.007), which means that the FM group reported significantly more worries in the PSWQ than the control group (*p=*.003), but not more worries than the EM group (*p=*.420).

**Table 3:** Results of the self report measures.

	Group			ANOVA
	Co	EM	FM	***F-*****test**
MIDAS (*M, SD)*	no headaches	14 (10)	46 (38)	p=.001 **
*severe disability*	no headaches	16%	67%	
BDI-II (*M, SD)*	6 (5)	10 (7)	12 (9)	p=.033 *
STAI-T (*M, SD)*	36 (9)	43 (11)	42 (12)	*p=.058*
PSWQ (*M, SD)*	39 (11)	46 (10)	49 (12)	p=.007 **

Groups were compared using one-way ANOVAs. * *p *< .05 ** *p *< .01

Co: control group; EM: episodic migraine group; FM: frequent migraine group

#### Task 1: Emotional Stroop test - words

**Figure 1:**
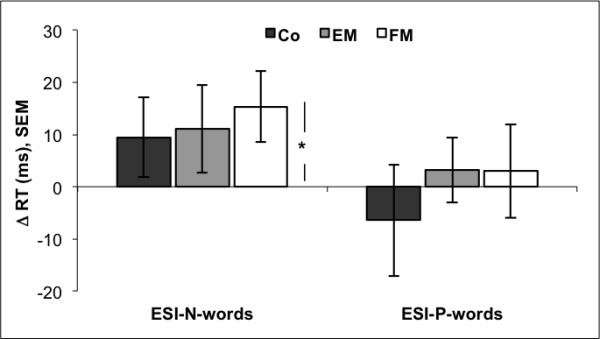
**Emotional Stroop indices for words**. Emotional Stroop indices for words comparing the color naming latencies of negative stimuli with the latencies of neutral stimuli (ESI-N) and comparing the response latencies of negative stimuli with the latencies of positive stimuli (ESI-P). ESI of all three groups are displayed (Co: control group, EM: episodic migraine group; FM: frequent migraine group). Asterisk indicates significant difference from zero for the FM group in ESI-N, ***p=***.043). One-way ANOVAs did not yield significant group effects for any of the two indices. RT: reaction time.

Figure 1 shows means and standard errors of the emotional Stroop indices ESI-N and ESI-P for words. Mean ESI-N of all three groups had positive values (controls: *M=*9.5 ms, *SEM *= 8; EM: *M=*12.5 ms, *SEM *= 8; FM: *M=*14.6 ms, *SEM *= 7), which implies that all three groups were slower in colour naming of negative than of neutral words. *T*-tests revealed a significant difference from zero only for the FM group (*t*(15) = 2.21, *p=*.043). ANOVA did not reveal a significant main effect of group, i.e. no group difference (*F*(2,49) = 0.11, *p = * .895). Mean ESI-P was slightly below zero for the control group and slightly positive for the EM and FM group (controls: *M=*-6.4 ms, *SEM *= 11; EM: *M=*4.3 ms, *SEM *= 6; FM: *M=*-0.4 ms, *SEM *= 9). *T*-tests did not reveal a significant difference from zero for any of the groups. There was no group difference, either (*F*(2,49)=.38, *p=*.687).

#### Task 2: Emotional Stroop test - faces

**Figure 2:**
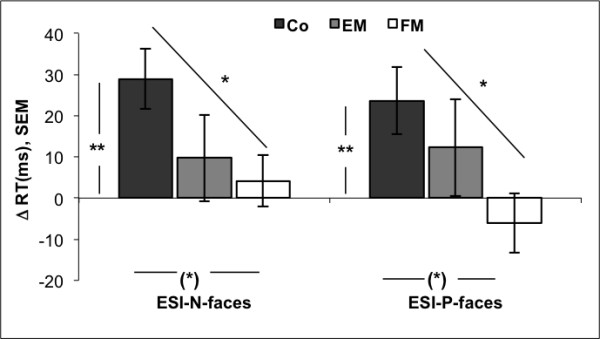
**Emotional Stroop indices for faces**. Emotional Stroop indizes for faces comparing the color naming latencies of negative stimuli with the latencies of neutral stimuli (ESI-N) and comparing the response latencies of negative stimuli with the latencies of positive stimuli (ESI-P). ESI of all three groups are displayed (Co: control group, EM: episodic migraine group; FM: frequent migraine group). Analyses of variances revealed a marginally significant group difference for ESI-P ((*) ***p***=.05), and for ESI-N ((*) ***p***=.075). For both indices there were significant differences between the Co and the FM group (ESI-N: ***p***=.038; ESI-P: ***p***=.016). The ESI-N of the controls differed significantly from zero (***t***(19) = 3.94, ***p=***.001), whereas the ESI-N of the migraine groups were not significantly different from zero. RT: reaction time.

The indices ESI-N and ESI-P for faces are displayed in Figure 2. The ESI-N for faces showed a linear decline from the control group to the FM group (controls: *M=*29.0 ms, *SEM *= 7; EM: *M=*7.7 ms, *SEM *= 11; FM: *M=*3.6 ms, *SEM *= 6). The ESI-N of the controls differed significantly from zero (*t*(19) = 3.94, *p=*.001), whereas the ESI-N of the EM and FM group were not significantly different from zero. This result indicates that the controls responded more slowly to negative faces than to neutral faces. Analysis of variances resulted in a trend toward a group effect (F(2, 50) = 2.73, *p=*.075). A priori set contrasts yielded a trend toward a difference between the ESI-N of the control group and the EM group (*p=*.076) and a significant difference between the control and the FM group (*p=*.038) with both migraine groups exhibiting lower indices than the controls. The results of the ESI-P for faces showed the same pattern: a linear decline from the control group to the FM group (controls: *M=*23.6 ms, *SEM *= 8; EM: *M=*11.2 ms, *SEM *= 12; FM: *M=*-9.92 ms, *SEM *= 7). The control group had the highest ESI-P, significantly above zero (*t*(19) = 2.91, *p=*.003) whereas the FM group had the lowest index, slightly below zero (*t*(15)=-1.36, *p=*.195), which means, the FM group responded faster to negative than to positive stimuli. The analysis of variances revealed a marginally significant group effect (*F*(2, 50) = 3.15, *p=*.05). A priori contrasts showed that the index of the control group was significantly higher than the ESI-P of the FM group (*p=*.016) but not higher than the ESI-P of the EM group (*p=*.35). This indicates that the group effect is due to the reaction differences of the control group and the FM group wheres the EM group reacted similar to the controls.

### Correlations

Table 4 displays the correlations of the indices with the self report scores and participants' characteristics. The word indices did not correlate with any of the headache parameters or self report scores. The face index ESI-N correlated significantly with migraine duration (*r=*-.341, *p=*.012). There was a trend to a correlation with headache frequency (*r=*-.228, *p=*.101). The ESI-P correlated significantly with headache frequency (*r=*-.305, *p=*.026) and marginally significantly with migraine duration (*r=*-.257, *p=*.064)

**Table 4:** Correlations of all indices with the headache parameters and self report scores.

	correlations			
	word indices		face indices	
	ESI-N	ESI-P	ESI-N	ESI-P
age	.201	.160	-.135	-.177
migraine duration	.187	.174	-.341 *	-.257 (*)
headache frequency	.050	.046	-.228 (*)	-.305 *
MIDAS	.037	.106	-.205	-.313 *
BDI-II	-.021	.078	-.218	-.097
STAI-T	-.105	.013	-.187	-.143
PSWQ	-.083	.098	-.242	-.185

* *p *< .05 (*) *p *< .10

ESI-N: Emotional Stroop index comparing the color naming latencies of negative stimuli with the latencies of neutral stimuli.

ESI-P: Emotional Stroop index comparing the color naming latencies of negative stimuli with the latencies of positive stimuli.

## Discussion and conclusions

According to the results from the self reports, the migraine patients in this study, especially the patients with very frequent and chronic migraine, resulted to be highly disabled by their headaches. Moreover, they reported more anxiety, depressive symptoms, and worries than the healthy control group.

In task 1, no group effects for the ESI-N were found despite a significant difference in color naming latencies of neutral and negative words in the FM group. All groups reacted the same way, with slower reactions to negative than to neutral words. No group effect was found for the index comparing the reaction times to negative words with the reaction times to positive words (ESI-P), either. Because of the missing group effects, the results cannot be interpreted in favor of the hypothesis of an attentional bias for negative words in the FM group.

One reason for the weak results in task one might lie in the small sample size. With low numbers like in the present study it is only possible to detect strong effects. Smaller but possibly meaningful differences do not become statistically significant. Moreover, already several former studies doubted the use of words as stimuli, because words might not be strong enough to elicit significant attentional biases, especially in pain patients [32-34]. Also, the words we used in the present study were general affective words. The use of words that reflect the common migraine triggers more specifically might lead to more pronounced effects.

In task 2, the controls showed a significant attentional bias to negative faces, whereas the FM group and the EM group did not. There was almost no difference between the reactions to neutral faces and the reactions to negative faces in the FM group, which was reflected by an index near zero. Comparing the color naming latencies for negative faces to those for positive faces, the effect became even stronger. The FM group here showed faster reactions to negative faces than to positive faces, with an index below zero, in contrast to the control group, which showed a high positive index.

The small indices of the FM patients, which in case of ESI-P is even negative, and the differences between FM patients and controls, could be an indicator of avoidance. Instead of resulting in the expected attentional bias towards negative social stimuli as reflected by angry faces, the subjects with FM showed unexpected avoidance behavior, i.e. they were faster in color naming for negative pictures than for neutral or positive pictures. Furthermore, an association of this avoidance with disease duration and migraine frequency, as reflected by significant correlations, could be found.

Our behavioral data support the self report data from a recent study. There, patients with chronic migraines reported significantly more avoidant coping strategies than patients with episodic migraines [35]. These findings are in line with the assumptions of Martin [36] and Martin and MacLeod [8], who stated that general avoidance of headache triggers might be a part of migraine worsening. They claimed that the role of avoidance in migraine exacerbation might be twofold: first, trigger avoidance does not decrease but rather increases the sensitivity towards the triggers, and therefore leads to an increased probability of migraine attacks in response to triggers [37]. Second, constantly avoiding situations that might possibly lead to migraine attacks may further result in loss of functional coping abilities in these situations and therefore may cause exactly the stress to the subjects they actually try to avoid [38]. Some findings of Martin and colleagues [38,39] examining the relationship between sensitivity and exposure to certain triggers like stress, noise or visual disturbances, support the idea of heightened trigger sensitivity with avoidance.

Our results indicate that the advice to avoid personal trigger factors in subjects with frequent migraines might lead to automated and maybe dysfunctional avoidance behavior in the long term. From the anxiety literature it is known that behavioral and cognitive avoidance play a role in the maintenance of disorders [40]. Clinicians should be made aware of the role dysfunctional avoidance behaviors could play in the process of migraine chronification.

As our study did not primarily aim to assess avoidance behavior, studies with psychological paradigms like the visual probe task, where both directions of attention can be investigated, would be needed to further strengthen this point. The relationship between trigger avoidance, dysfunctional coping strategies and disability in migraine patients merits further investigation.

## Competing interests

The authors declare that they have no competing interests.

## Authors' contributions

AP conceived of the study, conducted the experiments and performed the statistical analyses. CS participated in the design of the study and its coordination and helped to draft the manuscript. All authors read and approved the final manuscript.

## Pre-publication history

The pre-publication history for this paper can be accessed here:

http://www.biomedcentral.com/1471-2377/11/141/prepub
